# Supplementary material for: HIVprotI: an integrated web based platform for prediction and design of HIV proteins inhibitors
Source: J Cheminform. 2018 Mar 9;10:12. doi: 10.1186/s13321-018-0266-y (PMC5845081; doi:10.1186/s13321-018-0266-y)
Supplement: Supplementary file 2 — Additional file 2. Source code of HIVProtI web server. [file 13321_2018_266_MOESM2_ESM.zip › HIVprotI_Source-code/hivprotI/weka-3-6-6/doc/weka/core/AttributeExpression.html]

AttributeExpression


---


|  |  |  |
| --- | --- | --- |
| **Overview** \| **Package** \| **Class** \| **Tree** \| **Deprecated** \| **Index** \| **Help** \| **Weka's home** | |  |
| **PREV CLASS**   **NEXT CLASS** | **FRAMES**    **NO FRAMES**     **All Classes** |  |
| SUMMARY: NESTED \| FIELD \| CONSTR \| METHOD | DETAIL: FIELD \| CONSTR \| METHOD |  |


---


## weka.core Class AttributeExpression

```
java.lang.Object
  weka.core.AttributeExpression
```

**All Implemented Interfaces:**: java.io.Serializable, RevisionHandler

---

``` public class AttributeExpression extends java.lang.Object implements java.io.Serializable, RevisionHandler ```

A general purpose class for parsing mathematical expressions
involving attribute values. Values can be provided in an array
or in an Instance. Values are accessed in the expression by
prefixing their index (starting at 1) with the character 'a'.

```
 Example expression: a1^2*a5/log(a7*4.0)
```

Supported opperators: +, -, \*, /, ^, log, abs, cos, exp, sqrt,
floor, ceil, rint, tan, sin, (, ).

**Version:**
:   $Revision: 5989 $

**Author:**
:   Mark Hall

**See Also:**: Serialized Form

---

**Constructor Summary**

`AttributeExpression()`


| **Method Summary** | |
| --- | --- |
| `void` | `convertInfixToPostfix(java.lang.String infixExp)`             Converts a string containing a mathematical expression in infix form to postfix form. |
| `void` | `evaluateExpression(double[] vals)`             Evaluate the expression using the supplied array of attribute values. |
| `double` | `evaluateExpression(Instance instance)`             Evaluate the expression using the supplied Instance. |
| `java.lang.String` | `getPostFixExpression()`             Return the postfix expression |
| `java.lang.String` | `getRevision()`             Returns the revision string. |
| `java.lang.String` | `toString()` |

**Methods inherited from class java.lang.Object**

`equals, getClass, hashCode, notify, notifyAll, wait, wait, wait`

**Constructor Detail**

### AttributeExpression

```
public AttributeExpression()
```


**Method Detail**

### convertInfixToPostfix

```
public void convertInfixToPostfix(java.lang.String infixExp)
                           throws java.lang.Exception
```

:   Converts a string containing a mathematical expression in infix form
    to postfix form. The result is stored in the vector m\_postfixExpVector

    :   **Parameters:**: `infixExp` - the infix expression to convert **Throws:**: `java.lang.Exception` - if something goes wrong during the conversion

---


### evaluateExpression

```
public double evaluateExpression(Instance instance)
                          throws java.lang.Exception
```

:   Evaluate the expression using the supplied Instance.
    Assumes that the infix expression has been converted to
    postfix and stored in m\_postFixExpVector

    :   **Parameters:**: `instance` - the Instance containing values to apply the expression to **Throws:**: `java.lang.Exception` - if something goes wrong

---


### evaluateExpression

```
public void evaluateExpression(double[] vals)
                        throws java.lang.Exception
```

:   Evaluate the expression using the supplied array of attribute values.
    The result is stored in the last element of the array. Assumes that
    the infix expression has been converted to postfix and stored in
    m\_postFixExpVector

    :   **Parameters:**: `vals` - the values to apply the expression to **Throws:**: `java.lang.Exception` - if something goes wrong

---


### getPostFixExpression

```
public java.lang.String getPostFixExpression()
```

:   Return the postfix expression

    :   **Returns:**: the postfix expression as a String

---


### toString

```
public java.lang.String toString()
```

:   **Overrides:**: `toString` in class `java.lang.Object`

---


### getRevision

```
public java.lang.String getRevision()
```

:   Returns the revision string.

    :   **Specified by:**: `getRevision` in interface `RevisionHandler`
    :   **Returns:**: the revision


---


|  |  |  |
| --- | --- | --- |
| **Overview** \| **Package** \| **Class** \| **Tree** \| **Deprecated** \| **Index** \| **Help** \| **Weka's home** | |  |
| **PREV CLASS**   **NEXT CLASS** | **FRAMES**    **NO FRAMES**     **All Classes** |  |
| SUMMARY: NESTED \| FIELD \| CONSTR \| METHOD | DETAIL: FIELD \| CONSTR \| METHOD |  |


---
